# Appendiceal Mucocele in Amyand’s Hernia: A Case Report

**DOI:** 10.7759/cureus.88200

**Published:** 2025-07-17

**Authors:** Sergio G Moreno Hernandez, Daniel Nazario Cruz, Luis Cruz Benitez, Rosa Martha Morales López, Arath A Zamarripa Olmedo, Pedro J Curi-Curi, Omar E Valencia-Ledezma

**Affiliations:** 1 Surgery, Hospital Regional De Alta Especialidad De Ixtapaluca, Ixtapaluca, MEX; 2 General Surgery, Hospital Regional De Alta Especialidad De Ixtapaluca, Ixtapaluca, MEX; 3 Surgery, Universidad Nacional Autónoma de México, Mexico City, MEX; 4 Pathology, Hospital Regional De Alta Especialidad De Ixtapaluca, Ixtapaluca, MEX; 5 Investigation, Hospital Regional De Alta Especialidad De Ixtapaluca, Ixtapaluca, MEX

**Keywords:** amyand's hernia, appendiceal mucocele, appendicitis, histopathology, peritoneal pseudomyxioma

## Abstract

We report a rare case of a 78-year-old male who presented with abdominal pain and a right groin mass. Imaging revealed an inguinoscrotal hernia involving part of the large intestine, leading to anatomical distortion. Intraoperatively, an appendix with mucinous dilation was discovered and resected; histopathology confirmed an appendiceal mucocele (AM). This rare combination presents diagnostic challenges and requires prompt surgical intervention to prevent complications such as pseudomyxoma peritonei.

## Introduction

Amyand's hernia (AH) is a form of inguinal hernia characterized by the appendix contained within the hernial sac [1,2]. It is a rare and infrequent condition with a prevalence of 0.12-1% of all inguinal hernia cases, regardless of the presence of appendicitis [3,4]. Epidemiologically speaking, this condition predominantly affects males, with a 3:1 male-to-female ratio, and is more frequent in infants than adults [5].

Clinically, this type of AH is indistinguishable from an incarcerated hernia [6]. The most common signs and symptoms are a mass in the inguinal region that progressively enlarges over time and groin pain described as "continuous" with a pulling or burning sensation. In contrast to incarcerated hernia, the most common symptoms are localized pain, tumor or lump in the inguinal region, abdominal distension, nausea, vomiting, fever, reddish or purple coloration, and signs of intestinal obstruction if there is involvement of intestinal loops [7,8], so the differential diagnosis should be performed with complementary imaging studies [9].

Appendiceal mucocele (AM) is a dilation of the cecal appendix caused by the accumulation of mucus, which can lead to obstruction, neoplasia, or infection [4].

The incidence of this condition associated with AH is even rarer, ranging from 0.07% to 0.13% of cases [7]. After appendectomy, the five-year survival rate for simple AM ​​is 91% to 100%, but drops to 25% for malignant AM [10]. The mortality rate for AH ranges from 14% to 30%.

Imaging studies such as ultrasound or computed tomography can guide the diagnosis. The most relevant findings are a distended lumen of the appendix filled with fluid, along with an abrupt narrowing of its junction with the cecum [8,11].

Briefly, there are four types of AM [12]: a) simple or retention mucocele, which occurs due to luminal obstruction (caused by fecaliths or fibrosis) that leads to mucus accumulation without epithelial proliferation and is usually benign [13]; b) mucosal hyperplasia, which is characterized by benign hyperplasia of the mucus-producing appendiceal epithelium and is usually benign [13]; c) low-grade mucinous neoplasia, which is neoplasia with low-grade dysplasia that produces mucus and can perforate and is considered to have uncertain behavior and is potentially malignant [12,14]; d) mucinous adenocarcinoma, which is infiltrating malignant neoplasia with abundant mucus production and has a high risk of pseudomyxoma peritonei [12,14].

Surgical management is the primary treatment of choice. Laparoscopic surgery can be performed in cases of mucocele, considering the risk of appendiceal rupture or pseudomyxioma peritonei (PMP), which has a 9% incidence and is characterized by peritoneal tumor deposits, mucinous ascites, compaction of the omentum, and ovarian involvement in women [9].

This presents a diagnostic challenge because it is a rare and atypical presentation. The diagnosis is typically made intraoperatively, and the presence of a mucocele alters management by requiring avoidance of appendiceal perforation to prevent the development of pseudomyxoma peritonei [1,4,8].

## Case presentation

A 78-year-old male patient with a 45-year history of type 2 diabetes mellitus treated with insulin glargine (20 IU in the morning and 15 IU at night), a 20-year history of systemic arterial hypertension managed with losartan 50 mg/day, and a prior surgical amputation of the second to fourth toes on both feet, presented with a two-day history of fever reaching 39°C, dyspnea on minimal exertion, and non-foul-smelling, transparent expectoration. He denied other respiratory or gastrointestinal symptoms. Despite self-medicating with paracetamol, there was no clinical improvement, and he sought medical attention, where a soft tissue infection of the toe stumps was identified. This was unsuccessfully managed with combined oral and parenteral antibiotics along with local wound care.

Abdominal pain was later added to the symptoms, so he was admitted to our medical center's emergency room. At admission, his blood pressure was 95/58 mmHg, heart rate 108 beats per minute, respiratory rate 22 per minute, and temperature 36.9°C. Abdominal examination revealed tenderness on superficial and deep palpation in the right hypochondrium and inguinal region, a distended globular abdomen, tympanic sounds on percussion, inaudible peristalsis, and no rebound tenderness. Laboratory analysis was performed (Table 1).

**Table 1:** Laboratory results

Test	Result	Reference value
Leukocytes	23.1 x 10^3^ u/L	3.56-10 .3 u/L
D-dimer	26852 ng/ml	Less than 500 ng/ml
Procalcitonin	0.89 ng/ml	Less than 0.1 ng/ml
Creatinin	4.01 mg/dl	0.5-0.9 mg/dl
Sodium	120 mmol/l	136-145 mmol/l

**Figure 1:**
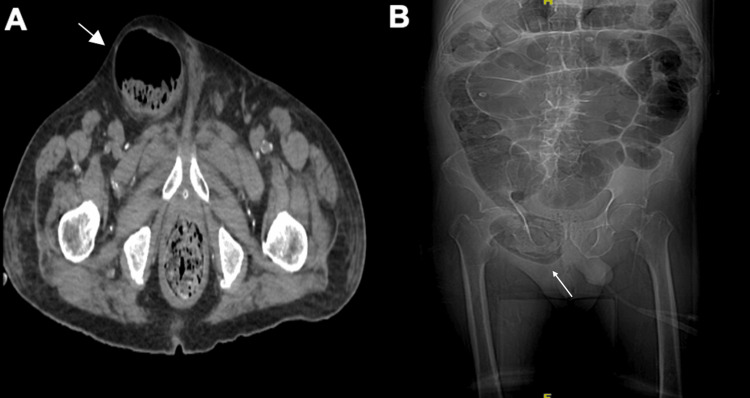
A. Axial abdominal computed tomography with Amyand's hernia (CT). B. Coronal CT scan demonstrating the hernial defect in the inguinal region.

An abdominal CT scan was performed (Figure 1), revealing a right inguinoscrotal hernia with a hernial sac containing fat and a cecal loop, causing traction of the ileocecal valve and retrograde dilation. The imaging also demonstrated an ascending colon loop with a diameter of 5.4 cm, a transverse colon measuring 6 cm, a cecum of 6.5 cm, and a jejunal loop measuring 2.6 cm. These findings were consistent with an incarcerated inguinal hernia associated with signs of intestinal obstruction.

**Figure 2:**
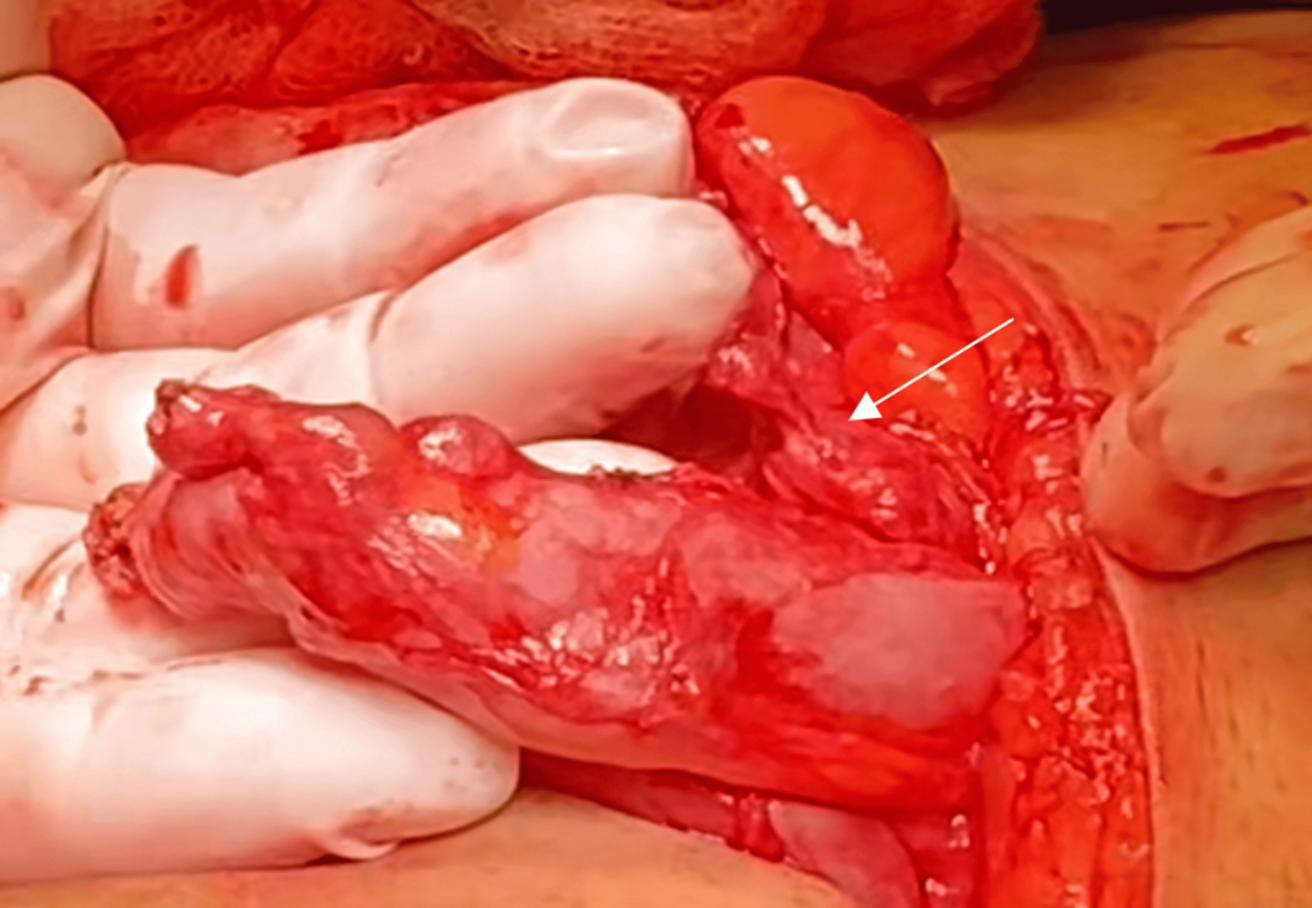
Inguinoscrotal hernia with a hernial sac containing the cecal appendix, showing signs of chronic appendicitis and no evidence of perforation.

Based on the imaging and laboratory findings, antibiotic therapy and analgesia were administered, and the patient was taken to the operating room for a right inguinal hernioplasty. An inguinoscrotal hernia was identified, with a 5 × 3 cm hernial ring and a 13 × 11 cm hernial sac containing a cecal appendix showing signs of chronic appendicitis, without evidence of perforation. An appendectomy was performed (Figure 2), with closure of the appendiceal stump using the Pouchot technique (Closure of the appendiceal stump with a loop without causing intussusception). The hernial sac was reduced into the inguinal canal, and a mesh was subsequently placed and secured to the pubic tubercle.

**Figure 3:**
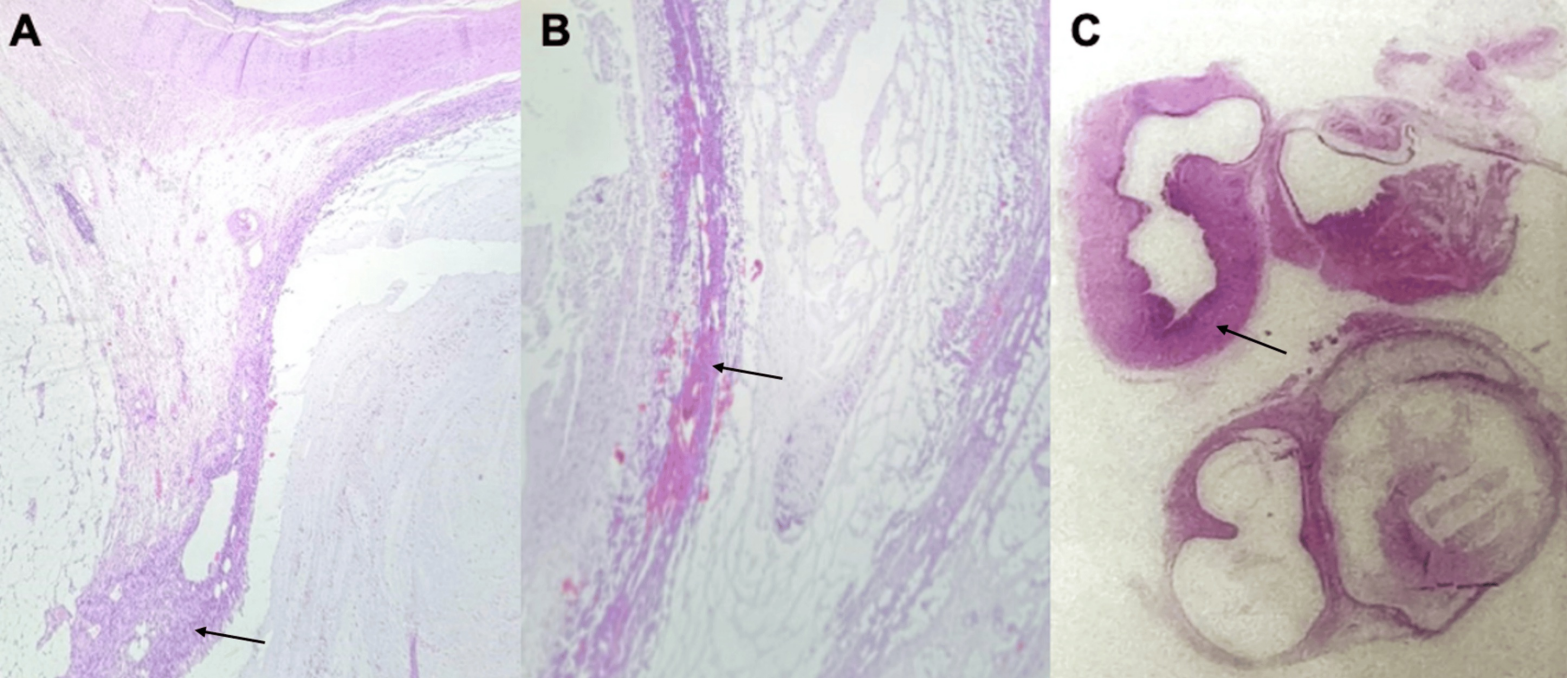
Appendiceal mucocele. A. Hematoxylin and eosin-stained photomicrograph at 40× showing a cystic formation lined by mucus-producing simple columnar epithelium. B. Hematoxylin and eosin-stained photomicrograph at 40× showing dilation of the appendiceal lumen, which is filled with mucus. C. Hematoxylin and eosin-stained photomicrograph at 4× showing a benign appendiceal neoplasm composed of a cystic dilation of simple epithelium.

The surgical specimen was sent to the pathology department, and the following pathological report was obtained: AM (Figure 3).

The patient presented with evidence of an inflammatory response six days after the surgical procedure, with no signs of recurrence, and was discharged from the hospital upon clinical improvement. He is currently being followed up in the outpatient clinic, with no signs of recurrence.

## Discussion

AH was first described and treated by Claude Amyand in 1735. The condition was later classified by Losanoff and Basson, who indicated that it could be managed either by reduction or appendectomy. The incidence of AH is rare, occurring in 0.19-1.7% of patients with inguinal hernia. When associated with appendicitis, its rarity increases even further, with an incidence ranging between 0.07% and 0.13% [5,6].

From a pathophysiological perspective, a systematic review suggested that the primary cause of appendicitis development in AH is external compression generated by muscle contraction and a transient increase in intra-abdominal pressure, leading to ischemia and a subsequent inflammatory process [11].

Patients presenting with signs and symptoms of incarceration or strangulation of an inguinal hernia can be initially assessed through physical examination. In the vast majority of cases, the diagnosis of AH is made intraoperatively. However, imaging, particularly computed tomography, has proven to be a valuable tool in suggesting the diagnosis [5], as demonstrated in this case.

Imaging studies such as ultrasound or computed tomography can assist in guiding the diagnosis, as in our clinical case. The most relevant findings include a distended appendix lumen filled with fluid, along with an abrupt narrowing at its junction with the cecum [8,11].

Nevertheless, even with imaging, the definitive diagnosis is typically established based on surgical findings and histopathological analysis [6], as occurred in our patient. The Losanoff and Basson classification (Table 2) is commonly used to guide management decisions. Treatment is well established for types 1, 3, and 4. Mesh repair is indicated for type 1 and contraindicated in types 3 and 4 [6].

**Table 2:** Classification of Losanoff and Basson Reference: [6]

Type	Description	Abdominal sepsis	Treatment	Use of mesh
1	Normal appendix inside the hernial sac	No	Reduction and placement	Yes
2	Acute appendicitis within the hernial sac	No	Appendectomy + primary hernia repair	Without mesh
3	Appendicitis within the inguinal hernial sac	Peritoneal or abdominal wall disease	Laparotomy + appendectomy + hernioplasty	Without mesh
4	As with type 3	Abdominal disease	Laparotomy + appendectomy + hernioplasty + treatment of concomitant disease	Without mesh

Specifically, as reviewed in the clinical case presented here, clinical and complementary studies revealed an incarcerated inguinoscrotal hernia associated with signs of intestinal obstruction. Therefore, surgery was performed, during which an AH was incidentally discovered, which, based on its intraoperative findings, was classified as grade 1 according to the Losanoff and Basson classification. The appendix was removed and sent for pathology, which revealed an AM.

## Conclusions

This case documents an exceptionally rare occurrence of an AM within an AH, highlighting the importance of considering such pathologies in atypical presentations of incarcerated inguinal hernias. The usefulness of imaging studies, particularly computed tomography, lies in their ability to suggest its presence preoperatively, allowing for appropriate surgical planning. Timely surgical management and histopathological evaluation are essential to prevent complications such as pseudomyxoma peritonei and to exclude malignancy. 

In this patient, the incidental finding of a cecal appendix within the hernial sac with signs of chronic inflammation and a subsequent histopathological diagnosis of AM highlights the importance of careful evaluation of the structures contained within an incarcerated hernia.

The Losanoff and Basson classification plays a fundamental role in surgical decision-making, as it establishes the indications for appendectomy, the type of hernia repair, and the use or omission of prosthetic material, depending on the condition of the appendix and the presence of abdominal sepsis. In this case, the absence of sepsis allowed for appendectomy and mesh placement, which, according to this classification, corresponds to type 1. Therefore, knowledge and application of this classification not only guide the safest and most effective surgical approach but also contribute to individualized management in complex scenarios, such as incidental findings of appendiceal pathology within inguinal hernias.

This decision prevented potential future complications and resulted in a satisfactory recovery for the patient.
